# Editorial: Women in science - regulatory science 2023

**DOI:** 10.3389/fmed.2024.1389438

**Published:** 2024-03-08

**Authors:** Mette Due Theilade Thomsen, Lisbeth Ehlert Knudsen

**Affiliations:** ^1^PIP Adviser, Lyngby, Denmark; ^2^Department of Public Health, University of Copenhagen, Copenhagen, Denmark

**Keywords:** digital health, connected health, regulatory harmonization, African Medicines Agency, joint assessment procedures, interviews, nanotechnology, Horizon scanning

The regulatory science call has received 5 publications from 2023 and 2024, all with female first or last authors, covering examples of methodological approaches to health empowerment and regulatory progress for medicine development.

Awad et al. describe the regulatory framework in the US, EU and China for connected health technology, which is the use of information technology, digital networks, artificial intelligence and machine learning to collect, share, and analyze data on individuals' health. Connected health technology involves the use of regulated medical devices, including software as a medical device, or consumer products, such as wearables or apps falling under regulatory discretion.

A need is underlined for streamlining, clarifying, and reinforcing the regulatory pathway for these new tools. The authors conclude on three critical areas where progress is warranted, which are optimization of the processes for validating new tools, an expansion of the regulatory workforce with advanced expertise in the new technologies and increasing knowledge-sharing among regulators to improve harmonization.

Qualitative research in clinical trial design is used to obtain a better understanding of the patient perspective and to include the voice of patients via a so-called patient-focused drug development. This has the long-term benefit of potentially optimizing the chances of later regulatory approval of the medicine, improving the outcome of Health Technology Assessment and bettering the chances of reimbursement of the medicine. Michel et al. present a literature review on the use of qualitative interviews conducted during clinical trials as part of a drug development program. The publication also reviews relevant regulatory guidelines and reports from health technology agencies and learned societies.

The review was done to understand the current practices for patient interviews in drug development, the methodology employed, and how data generated from such interviews are considered by health authorities for marketing authorization and reimbursement. The qualitative data identified in the literature search was found to provide useful and important information on a variety of parameters, and data from labels and health technology assessments identified in this review demonstrate that qualitative data can play an important role in approval processes. The authors conclude by expressing a need for guideline development addressing how to optimize in-trial interviews for the purpose of improving the conditions for a future marketing authorization and reimbursement.

Unwarranted extended hospital stays increase the risk of morbidity and mortality associated with the stay, meaning that the longer your hospital stay, the higher the risk of hospital-acquired complications, morbidity, and overall mortality. To assess the quality of risk prediction models for hospital extended length of stay (LOS) and identify validated prediction variables for the risk of prolonged LOS in hospital admissions, Gokhale et al. conducted a systematic review and meta-analysis of studies including statistical and machine learning methods. The most frequently used variables used to predict prolonged LOS were risk scores associated with severity of illness, demographic and anthropometric variables and admission characteristics.

Both machine learning and statistical modeling demonstrated good predictive performance, but models were often not externally validated and had poor overall study quality. The authors recommend future studies to improve data quality by adoption of guidelines and external validation.

Nanotechnology-enabled health products (NHPs) are nanomedicines, i.e., medicinal products including nanomaterials, and nanomedical devices, i.e., medical devices including nanomaterials. Rodríguez-Gómez et al. address the need for regulatory guidelines for NHPs by use of so-called Horizon scanning in literature, patents and documents from regulatory agencies. Horizon scanning methodology is a practice used among legislators and health authorities for anticipating future regulatory needs on specific topics in the short to medium term.

The authors have developed a methodology for predicting which nanotechnology products will trend in the future, and based on that, they analyze the current regulatory landscape and evaluate on the need for developing up-to-date regulatory guidelines. The results of the Horizon scanning show a clear trend toward development of drug delivery systems as well as a trend in the development of nanomaterials for dental applications such as surface filling or tooth replacement.

The authors conclude that a continued focus on developing robust and adaptable regulatory guidelines for NHPs is of utmost importance for facilitating safe and effective transition of such products from the research stage to clinical application.

Finally, Miletic et al. present in an interesting and well-elaborated review the perspective of the International Federation of Pharmaceutical Manufacturers and Associations (IFPMA) Africa Regulatory Network (ARN) on how to achieve an efficient evaluation of regulatory applications and better access to new medicines in Africa. The authors describe the results of a survey conducted with innovative biopharmaceutical companies on experiences using regional joint assessment procedures (JAPs) in Africa. Marketing authorization for new medicines in Africa can be obtained via international procedures such as WHO Collaborative Registration Procedures (CRP), Swissmedic's Marketing Authorisation for Global Health Products (MAGHP) and EU Medicines for All (EU-M4ALL), or they can be obtained via one of three African regional joint assessment programs. The review describes the procedures as well as the obstacles still existing with using these collaborative regional programs, such as long assessment times, national necessary procedures after the initial assessment, insufficient resource allocation etc. These obstacles call for harmonization of requirements across countries and regions. Such improvements will work positively toward establishing of the African Medicines Agency (AMA) in the future.

The five submitted publications cover scientific methodological issues calling for regulatory actions such as more methodological approaches to factors important to length of stay in hospitals, use of qualitative data from patients to improve drug development and approval, use of connected health approaches globally, guidelines for the registration of nanotechnology-enabled health products and finally the inclusion of Africa as equal partner in drug development.

## Author contributions

MT: Writing – review & editing, Writing – original draft. LK: Writing – review & editing, Writing – original draft.

